# Added value of multiphase CTA imaging for thrombus perviousness assessment

**DOI:** 10.1007/s00234-017-1907-y

**Published:** 2017-09-30

**Authors:** E. M. M. Santos, C. D. d’Esterre, K. M. Treurniet, W. J. Niessen, M. Najm, M. Goyal, A. M. Demchuk, C. B. Majoie, B. K. Menon, H. A. Marquering, PRove-IT investigators, Jennifer Mandzia, Enrico Fainardi, Marta Rubiera, Alexander V. Khaw, Andrea Zini, JJ. Shankar

**Affiliations:** 1 Department of Biomedical Engineering and Physics, Academic Medical Center, Amsterdam, The Netherlands; 2 Department of Medical Informatics, Erasmus Medical Center, Rotterdam, The Netherlands; 3 Departments of Neurosciences, Radiology and Community Health Sciences, University of Calgary, Calgary, Canada; 4 Department of Radiology and Nuclear Medicine, Academic Medical Center, Amsterdam, The Netherlands; 5 Faculty of Applied Sciences, Delft University of Technology, Delft, The Netherlands

**Keywords:** Acute ischemic stroke, Thrombus characteristics, Thrombus permeability, CT multi-phase, CTA

## Abstract

**Purpose:**

Thrombus perviousness has been associated with favorable functional outcome in acute ischemic stroke (AIS) patients. Measuring thrombus perviousness on CTA may be suboptimal due to potential delay in contrast agent arrival in occluded arteries at the moment of imaging. Dynamic sequences acquired over time can potentially overcome this issue. We investigate if dynamic CTA has added value in assessing thrombus perviousness.

**Methods:**

Prospectively collected image data of AIS patients with proven occlusion of the anterior or posterior circulation with thin-slice multi-phase CTA (MCTA) and non-contrast CT were co-registered (*n* = 221). Thrombus attenuation increase (TAI; a perviousness measure) was measured for the arterial, venous, and delayed phase of the MCTA and time-invariant CTAs (TiCTA). Associations with favorable clinical outcome (90-day mRS ≤ 2) were assessed using univariate and multivariable regressions and calculating areas under receiver operating curves (AUC).

**Results:**

TAI determined from the arterial phase CTA was superior in the association with favorable outcome with OR = 1.21 per 10 HU increase (95%CI 1.04–1.41, AUC 0.62, *p* = 0.014) compared to any other phase (venous 1.14(95%CI 1.01–1.30, AUC 0.58, *p* = 0.033), delayed 1.046(95%CI 0.919–1.19, AUC 0.53, *p* = 0.50)), and TiCTA (1.15(95%CI 1.02–1.30, AUC 0.60, *p* = 0.022). In the multivariable model, only TAI on arterial phase was significantly associated with favorable outcome (aOR 1.59, 95%CI 1.04–2.43, *p* = 0.032).

**Conclusion:**

Association between TAI with functional outcome was optimal on arterial-phase CTA such that dynamic CTA imaging has no additional benefits in current thrombus perviousness assessment, thereby suggesting that the delay of contrast arrival at the clot is a key variable for patient functional outcome.

## Introduction

Assessing thrombus characteristics on admission imaging may assist in acute ischemic stroke (AIS) management [1]. Thrombus length, density, and burden assessed on acute imaging has been associated with patient functional outcome and treatment success [2, 3]. Thrombus permeability is also considered to be associated with improved outcomes. Since the permeability of thrombi cannot be assessed in standard radiological imaging, derived metrics have been introduced such as attenuation increase (TAI) and void fraction. To distinguish the permeability from these derived measures, these characteristics have been referred to as thrombus perviousness measures [1]. TAI compares thrombus attenuation on CTA with NCCT and has been shown to be strongly associated with intravenous plasminogen activator treatment success, favorable functional outcome, and admission deficit in AIS patients [4, 5]. A potential drawback of this CTA-based measure is that enhancement of arteries (and thrombus) on CTA is affected by the timing of imaging [6]. In occluded arteries, with or without a permeable thrombus, arterial filling proximal and distal to a blood clot is delayed compared to the contralateral healthy side. Therefore, thrombus permeability assessment may be suboptimal if the CTA is acquired before contrast arrival in the occluded artery. A dynamically acquired CTA has the potential to resolve this timing issue. Furthermore, dynamically acquired CTA allows the generation of a time-invariant CTA in the form of a temporal maximum intensity profile (TiCTA) [6–10]. Recently, a multiphase CTA (MCTA) imaging protocol was developed in which multiple temporal image data sets are generated with a high spatial resolution, which also allows the generation of TiCTA [11]. We hypothesize that TAI imaged with MCTA is potentially stronger associated with good functional outcome and admission deficit in patients with AIS than a “snapshot” CTA.

## Material and methods

### Patient inclusion

We retrospectively collected clinical and image data of 299 patients with proven occlusion of the anterior and posterior circulation from the “Measuring Collaterals With Multi-phase CT Angiography in Patients With Ischemic Stroke” (PRove-IT) trial population (protocol at https://clinicaltrials.gov/ct2/show/NCT02184936). Patient exclusion criteria for this substudy were absence of functional outcome data (*n* = 5), incomplete axial coverage (*n* = 6), excessive noise (*n* = 2), movement (*n* = 45) and cerebrospinal fluid shunt (*n* = 1) induced artifacts, and insufficient contrast on the arterial-phase CTA to accurately perform the thrombus measurements (*n* = 8). This resulted in 233 patients that were included in this study. The Conjoint Health Research Ethics Board approved the study.

### Imaging

**Fig. 1:**
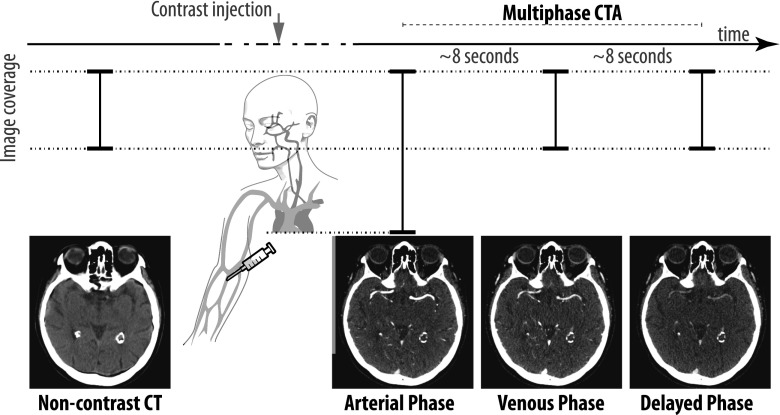
Graphical illustration of the non-contrast and multiphase CT angiography imaging protocol. From left to right: all patients underwent standard non-contrast CT (5-mm section thickness) followed by a multiphase CT angiography (0.625-mm section thickness). The multiphase CT angiography is performed in three phases after 80 mL contrast material injection without reinjections. The first phase: the “arterial phase” is timed to occur during the peak arterial phase and is triggered by bolus monitoring. The next two, the “Venous phase” and the “Delayed phase” are performed, the acquisition starts 7–9 s apart after the arrival of contrast to the descending aorta. Coverage of the non-contrast CT and of the venous-phase and delayed-phase CT angiography (CTA) are from the skull base to the vertex. The arterial-phase CTA covers the aortic arch to the vertex

In the PRove-IT study population, all patients underwent standard unenhanced CT with 5-mm section thickness followed by a 0.625-mm section thickness multiphase CT angiography (Figure 1) [11]. MCTA techniques generate time-resolved cerebral angiograms of brain vasculature from the skull base to the vertex in three phases after 80 mL contrast material injection (68% ioversol, Optiray 320; Mallinckrodt, St Louis, Mo) at a rate of 5 mL/s and followed by a 50-mL normal saline chase at a rate of 6 mL/s. The mean estimated effective dose was 5.0 ± 0.5 mSv for the first phase CTA and 1.0 ± 0.5 mSv per additional phase of the multiphase CT angiography. The first phase covers the aortic arch to the vertex and is timed to scan during the peak arterial phase in a healthy brain (bolus monitoring triggering). The next two phases are performed from the skull base to the vertex without injection of new contrast about 7–9 s apart as follow: the first delayed image acquisition starts after the arrival of contrast in the descending aorta. The second delayed imaging starts in the late venous phase. With this acquisition protocol, we obtain an image for three phases, which will be referred to as arterial, venous, and delayed phase respectively in the remainder of this manuscript.

From the MCTA data, a TiCTA was generated as a temporal maximum intensity projection of the three phases [9]. To avoid artifacts due to motion between the acquisition phases, each phase was co-registered [12] with the arterial phase using the registration software Elastix [13].

### Thrombus perviousness

The TAI measurements were performed as previously described [14]: Three spherical regions of interests (radius = 1 mm) were placed in the thrombus on the arterial-phase CTA. The placement of the region of interests was supported by a custom-developed interface in Mevislab [15] in which NCCT and the arterial phase CTA were simultaneously displayed. The assessments were performed by one of the two trained raters (ES or KT) and an expert rater (CM) if the other raters were unsure of their assessment due to a poor image enhancement. Initially, the arterial, the venous, and the delayed phase CTAs and NCCT images were co-registered with Elastix [13] using a rigid registration method. If the automated registration was suboptimal, a manual correction was performed (ES) using a toolbox available in Mevislab [15]. Using the co-registered images, the manually placed regions of interests in the arterial-phase CTA were automatically projected on all other CT images.

The mean attenuation of the manually placed regions of interests were used to calculate four TAI measures: the TAI_Arterial_, TAI_Venous_, TAI_Delayed_, and TAI_TiCTA_ by subtracting the thrombus attenuation on NCCT from the attenuation on the arterial, venous, and delayed phase CTAs and TiCTA, respectively.

### Statistical analysis

The association of the four TAI measures with functional outcome was assessed by performing univariate regression analysis. Strength of these associations were compared using the area under the receiver operating characteristic curve. Favorable outcome was defined as a score of 2 or less on the modified Rankin Scale (mRS) at 90 days. Functional deficit on admission was scored with the National Institutes of Health Stroke Scale (NIHSS), which was considered continuous in our analyses.

Data is presented with mean and standard deviation for normally distributed values or with median and interquartile range (IQR) otherwise. Normality of the distributions was assessed using the Shapiro-Wilk test. Categorical and ordinal data were presented using count and proportions. TAI outliers (*n* = 10) were eliminated using the Tukey outlier filter method [16]. Differences between the four TAI measures were assessed using pairwise comparisons (the Student test or the Wilcoxon signed-rank test).

#### Association of TAI with favorable functional outcome

TAI differences between patients with favorable and unfavorable outcome were assessed using the Kruskal–Wallis test. The odds ratio (OR) with 95% confidence intervals (95%CI) to favorable outcome per 10 HU increase in TAI were calculated using univariate logistic regressions. Models were compared using information theory approaches by calculating the Akaike information criterion (AIC) and Bayesian information criterion (BIC).

#### Association of TAI with admission deficit

Spearman’s correlation coefficient and the fit of the regression *R* [2] were calculated to assess the association between TAI measures and baseline NIHSS. Linear regression models describing the association between the four TAI measures and baseline NIHSS were compared using AIC and BIC.

#### Multivariable analysis

Baseline clinical variables with statistically significant associations with favorable outcome and TAI were included in a multivariable logistic regression analysis. Association of baseline clinical variables with favorable outcome was evaluated using logistic regression. The assessment of the association of baseline clinical variables with the TAI measures was performed using one-way Kruskal–Wallis ANOVA for ordinal variables, Spearman’s correlation coefficient for non-parametrically distributed variables, fit of the regression *R* [2] for parametrically distributed variables, and the Wilcoxon signed-rank test for dichotomized data.

Statistical significance was set to a *p* value < 0.05 for all analyses. Analyses were performed using IBM SPSS Statistics software, version 21.0 (IBM Corporation, Armonk, NY, USA).

## Results

TAI measures distributed for baseline and outcome characteristics are presented in Table 1. The average age was 71 (± 13) years, 51% (121) of the patients were female and the median admission National Institutes of Health Stroke Scale (NIHSS) was 13 (IQR 6–19). The majority (59.1%) of the patients had a favorable functional outcome. Nine (3.8%) patients required additional manual correction of the image registration. Median TAIs were 37 (IQR 28–49), 35 (IQR 27–51), 30 (IQR 20–42), and 50 (IQR 40–70) HU respectively for the arterial, the venous, and the delayed phase and the TiCTA. All but the TAI from the arterial and the venous phase CTAs were significantly different. Associations of baseline characteristics with functional outcome are presented in Table 2.

**Table 1:** TAI measures distributed for baseline clinical data and their associations with continuous clinical baseline and outcome variables

Dichotomized and ordinal variables						
		*n* (%)	TAI median(IQR) in HU			
			Arterial-phase	Venous-phase	Delayed-phase	TiCTA
All patients		221(100%)	36.7(28.0–48.5)	35(26.6–50.7)	30.1(20.2–41.6)	50.5(40.3–70.1)
Sex	Female	111(50.2%)	39.7(30.7–52.9)	37.1(29.5–51.9)	31.4(22.4–43.6)	53.2(43.4–72.1)
	Male	110(49.8%)	34.1(25.3–44.8)	33.4(24.7–45.9)	27.1(19.8–39.9)	47.7(37.9–64.4)
Occlusion location	ICA	14(6.3%)	29.9(21.2–37.9)	35.6(21.7–51.6)	22.1(16.8–41.5)	45.2(35.1–77.6)
	M1	94(42.5%)	34.9(26.0–45.4)	34.4(26.9–48.3)	31.1(22.4–44.1)	49.0(39.5–69.8)
	M2	67(30.3%)	38.4(30.9–52.9)	39.1(28.1–58.6)	33.5(22.7–49.0)	56.0(43.1–73.3)
	M3 and M4	18(8.1%)	38.8(33.1–56.0)	33.4(23.0–64.6)	23.4(16.8–34.3)	48.5(38.9–78.5)
	Basilar	6(2.7%)	15.9(8.2–50.5)	13.6(7.3–21.6)	10.9(8.6–13.1)	26.3(23.6–56.1)
	PCA	17(7.7%)	43.9(33.4–61.7)	38.0 (30.9–43.0)	23.8(16.9–34.0)	51.6(47.5–67.9)
	ACA	5(2.3%)	41.5(35.2–41.5)	28.4(21.0–32.7)	34.4(10.0–36.9)	49.8(44.5–50.7)
Treated with anithrombolitics	no	159(73.3%)	35.1(25.3–44.9)	34.2(25.6–47.7)	28.4(19.0–41.6)	49.4(38.1–66.3)
	yes	58(26.7%)	42.1(31.3–57.8)	36.6(28.5–71.7)	33.2(23.1–45.4)	56.1(43.8–80.0)
Treatment decision	None	49(22.3%)	41.3(31.2–57.5)	37.6(32.2–64.6)	33.0(23.1–40.5)	54.0(43.9–78.5)
	IV-TPA	82(37.3%)	37.8(27.8–48.1)	34.5(26.9–51.1)	29.5(20.6–42.7)	51.4(38.9–68.7)
	IA-TPA	25(11.4%)	33.9(29.4–42.8)	35.6(26.7–65.9)	26.6(20.2–50.7)	47.1(38.4–81.6)
	IV + IA-TPA	58(26.4%)	34.8(24.2–42.8)	33.8(25.4–42.4)	28.6(17.9–41.6)	47.1(38.3–57.9)
	Tenecteplase	6(2.7%)	40.3(32.7–43.9)	35.9(25.8–42.4)	27.1(15.9–41.4)	49.9(40.9–54.2)
mRS score	0	40(18.1%)	38.0(26.5–47.1)	36.3(24.2–51.7)	25.0(18.3–46.2)	50.9(38.4–77.9)
	1	48(21.7%)	40.0(31.5–57.7)	34.5(27.6–53.2)	34.3(20.3–50.6)	57.7(45.5–72.9)
	2	43(19.5%)	41.5(30.9–52.8)	37.1(29.5–58.6)	28.6(20.7–43.4)	49.7(43.4–72.4)
	3	24(10.9%)	34.8(26.9–57.0)	34.6(24.7–45.7)	30.8(22.4–42.0)	52.1(36.1–78.4)
	4	25(11.3%)	30.9(24.0–37.5)	33.5(26.0–41.4)	29.4(16.6–37.6)	45.5(38.1–64.4)
	5	6(2.7%)	36.2(29.7–47.0)	40.6(28.8–53.0)	32.6(30.3–34.8)	52.1(39.9–60.0)
	6	35(15.8%)	33.2(20.9–42.2)	33.4(20.7–41.9)	26.4(19.8–40.5)	43.9(31.1–56.0)
Functional outcome	Unfavorable	90(40.7%)	33.3(24.7–42.8)	33.5(24.7–44.0)	30.3(20.2–39.7)	46.1(36.8–63.4)
	Favorable	131(59.3%)	39.5(30.9–51.0)	35.6(27.6–54.0)	30.0(20.2–44.5)	52.7(42.6–73.3)
Continuous variables						
	Unit	Median(IQR)	Spearman’s rho (linear regression beta’s per 10HU)			
			Arterial phase	Venous phase	Delayed phase	TiCTA
Age	Year	73 (62–80)	0 (.013)	.133* (.046)	.162* (.082)	.089 (.037)
Hematocrit	%	42 (38–45)	.009 (0)	−.034 (0)	−.026 (0)	−.006 (0)
NIHSS	Score	12 (6–19)	−.229**(−.087)	−.099 (−.033)	−.036 (−.01)	−.161*(−.044)

mRS score: 0, no symptoms at all; 1, no significant disability despite symptoms; 2, slight disability; 3, moderate disability; 4, moderately severe disability; 5, severe disability; 6, dead

*TAI* thrombus attenuation increase, *HU* Hounsfield unit, *IQR* interquartile range, *TiCTA* time invariant computed tomography angiography, *IV-rtPA* intravenous recombinant tissue-type plasminogen activator treatment, *IA-TPA* intra-arterial recombinant tissue-type plasminogen activator treatment, *mRS* modified Rankin Scale, *NIHSS* National Institutes of Health Stroke Scale, *ACA* anterior cerebral artery, *ICA* intracranial cerebral artery, *M1* sphenoidal segment of the middle cerebral artery, *M2* insular segment of the middle cerebral artery, *M3* opercular segment of the middle cerebral artery, *M4* cortical segment of the middle cerebral artery, *PCA* posterior cerebral artery

**Correlation is significant at the 0.01 level; *Correlation is significant at the 0.05 level

**Table 2:** Baseline and follow-up clinical data, descriptive analysis and their associations with Functional outcome

Dichotomized and ordinal variables						
		All patients *n* (%)	Functional outcome		Association with favorable outcome	
			Favorable *n* (%)	Unfavorable *n* (%)	OR (95%CI)	*p*
All patients		221(100%)	131(59.3%)	90(40.7%)		
Sex	Female	111(50.2%)	64(48.9%)	47(52.2%)	0.6(0.3–1.3)	0.20
	Male	110(49.8%)	67(51.1%)	43(47.8%)		
Occlusion location	ICA	14(6.3%)	8(6.1%)	6(6.7%)	4.6(0.4–51.0)	0.21
	M1	94(42.5%)	45(34.4%)	49(54.4%)	4.1(0.5–31.9)	0.18
	M2	67(30.3%)	45(34.4%)	22(24.4%)	3.3(0.4–25.0)	0.25
	M3 and M4	18(8.1%)	16(12.2%)	2(2.2%)	7.0(0.6–87.3)	0.13
	Basilar	6(2.7%)	4(3.1%)	2(2.2%)	2.7(0.1–51.3)	0.51
	PCA	17(7.7%)	11(8.4%)	6(6.7%)	2.7(0.3–26.6)	0.39
	ACA	5(2.3%)	2(1.5%)	3(3.3%)		
Treated with antithrombolitics	no	159(73.3%)	92(71.3%)	67(76.1%)	0.4(0.1–2.0)	0.29
	yes	58(26.7%)	37(28.7%)	21(23.9%)		
Treatment	None	49(22.3%)	30(22.9%)	19(21.3%)	0.3(0.0–5.6)	0.44
	IV-TPA	82(37.3%)	47(35.9%)	35(39.3%)	1.4(0.1–15.6)	0.78
	IA-TPA	25(11.4%)	13(9.9%)	12(13.5%)	1.7(0.1–22.2)	0.69
	IV+IA-TPA	58(26.4%)	36(27.5%)	22(24.7%)	4.6(0.4–59.2)	0.24
	Tenecteplase	6(2.7%)	5(3.8%)	1(1.1%)		
Continuous variables						
	Unit	Median(IQR)	Functional outcome		Association with favorable outcome	
			Favorable	Unfavorable	OR (95%CI)	*p*
Age	Year	73 (62–80)	*71(58–77)*	*77(69–84)*	*0.96(0.93–0.98)*	*0.002*
Hematocrit	%	0.42 (0.38–0.45)	0.42(0.40–0.45)	0.41(0.36–0.45)	11.2(0.0–6405.7)	0.46
NIHSS	score	12 (6–19)	*9(5–15)*	*18(12–23)*	*0.82(0.77–0.88)*	*<0.001*

*TAI* thrombus attenuation increase, *HU* Hounsfield unit, *OR* odds ratio, *CI* confidence interval, *p* significance value, *IQR* interquartile range, *TiCTA* time invariant computed tomography angiography, *IV-rtPA* intravenous recombinant tissue-type plasminogen activator treatment, *IA-TPA* intra-arterial recombinant tissue-type plasminogen activator treatment; *mRS* modified Rankin Scale, *NIHSS* National Institutes of Health Stroke Scale, *ACA* anterior cerebral artery, *ICA* intracranial cerebral artery, *M1* sphenoidal segment of the middle cerebral artery, *M2* insular segment of the middle cerebral artery, *M3* opercular segment of the middle cerebral artery, *M4* cortical segment of the middle cerebral artery, *PCA* posterior cerebral artery mRS score: 0, no symptoms at all; 1, no significant disability despite symptoms; 2, slight disability; 3, moderate disability; 4, moderately severe disability; 5, severe disability; 6, dead; and for italic values, *p* is significant at the 0.05 level

### TAI association with clinical outcome

**Fig. 2:**
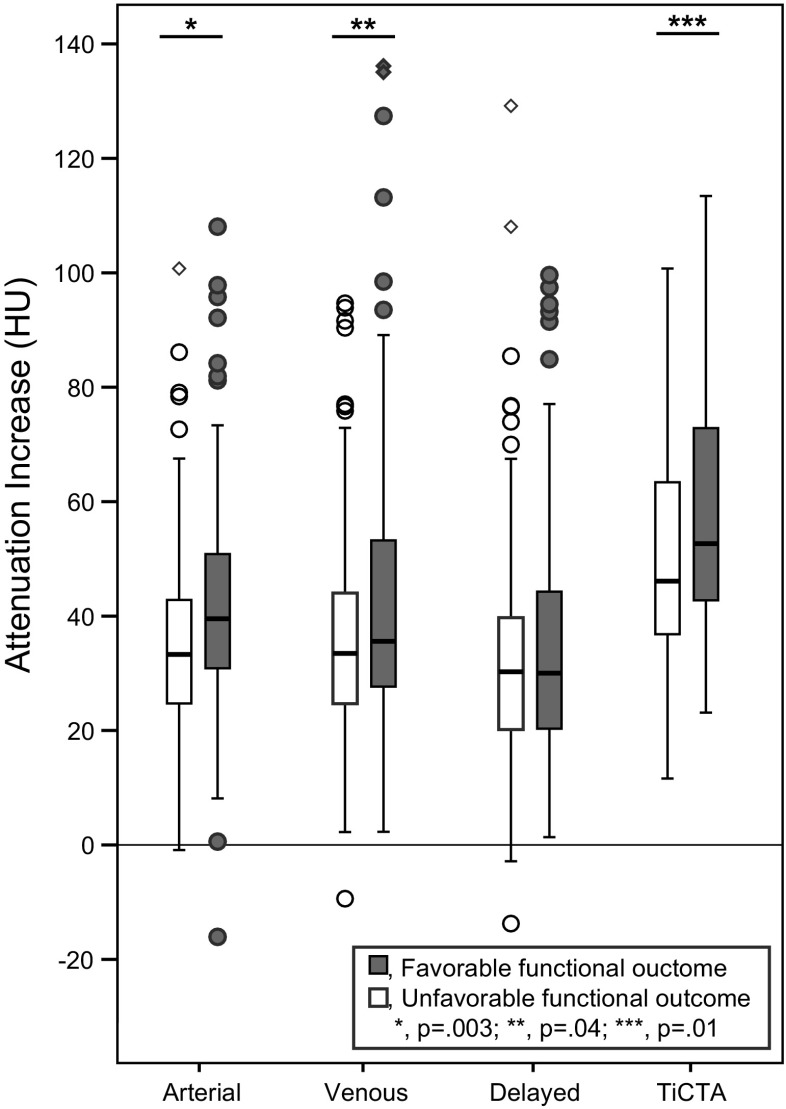
Boxplot of the TAI measures on arterial-phase, venous-phase, delayed-phase CTA, and on TiCTA for patients with unfavorable (mRS > 2) and favorable (mRS ≤ 2) functional outcome. There were statistically significant differences in the distributions of perviousness between the two groups for the TAI assessed on arterial-phase, venous-phase, and on TiCTA (Kruskal–Wallis Test). HU indicates Hounsfield Unit; CTA, computed tomography angiography; TiCTA, time invariant computed tomography angiography; p, significance value; mRS, modified Rankin Scale; mRS score: 0, no symptoms at all; 1, no significant disability despite symptoms; 2, slight disability; 3, moderate disability; 4, moderately severe disability; 5, severe disability; 6, dead

The TAI measures were significantly different between patients with favorable and unfavorable functional outcome for the arterial-phase CTA, the venous-phase CTA, and TiCTA (*p* = 0.003, *p* = 0.044, and *p* = 0.011, respectively). The TAI distributions per outcome group are shown in Fig. 2. In the univariate models, the ORs (95% CI, *p*) for a favorable outcome per 10 HU increase in TAIs were 1.21 (95%CI 1.04–1.41, *p* = 0.014) for TAI_Arterial_, 1.14 (95%CI 1.01–1.30, *p* = 0.033) for TAI_Venous_, and 1.15 (95%CI 1.02–1.30, *p* = 0.022) for TAI_TiCTA_. There was no significant association between TAI_Delayed_ and favorable outcome. The AUCs of the receiver operating curves were 0.62, 0.58, 0.53, and 0.60, respectively for the arterial phase, the venous phase, the delayed phase, and the TiCTA. The AIC and BIC were both lowest for TAI_Arterial_ (Table 3).

**Table 3:** Association of TAI measures with outcomes using logistic regression analysis receiver operating curve analysis, linear regression and Akaike and Bayesian information criterion

	Clinical outcome					Admission deficit			
	Univariate logistic regression analysis					Linear regression			
10 HU increase of TAI	OR (95% CI)	*p*	AUC	AIC	BIC	B (95% CI)	*p*	AIC	BIC
Arterial phase	*1.21(1.041–1.414)*	*0.01*	0.62	296.1	302.9	*−0.87(−1.4 to − 0.34)*	*0.002*	903.0	909.7
Venous phase	*1.148(1.011–1.303)*	*0.03*	0.58	297.8	304.6	−0.33(−0.78 to 0.11)	0.139	910.9	917.7
Delayed phase	1.046(0.919–1.19)	0.50	0.53	302.3	309.1	−0.10(−0.59 to 0.39)	0.681	913.0	919.7
TiCTA	*1.153(1.021–1.301)*	*0.02*	0.60	297.0	303.8	*−0.44(−0.87 to − 0.01)*	*0.044*	909.0	915.8

*TAI* thrombus attenuation increase, *OR* odds ratio, *aOR* adjusted odds ratio, *CI* confidence interval, *B* linear regression coeficient, *AUC* area under the curve, *AIC* Akaike information criterion, *BIC* Bayesian information criterion, *p* significance value, *HU* Hounsfield unit, *TiCTA* time invariant computed tomography angiography; and italic *p* is significant at the 0.05 level

### TAI association with admission deficit

Baseline NIHSS was significantly correlated with TAI_Arterial_ and TAI_TiCTA_ with a Spearman’s correlation coefficient and *R* [2] of − .229 (.045) and − .161(.044), respectively (Table 1). From the linear regression, the beta coefficients were −0.87 (95%CI − 1.40 to − 0.34, *p* = 0.002) and − 0.44 (95%CI − 0.87 to − 0.01, *p* = 0.044) per 10 HU increase of TAI_Arterial_ and TAI_TiCTA_, respectively. There was no significant association between TAI_Delayed_ and TAI_Venous_ and baseline NIHSS. The AIC and BIC were lowest for TAI_Arterial_ (Table 3).

### Multivariable analysis

Of all clinical variables, only age and baseline NIHSS were significantly associated with favorable outcome (Table 2). Baseline NIHSS was significantly correlated with TAI_Arterial_ and TAI_TiCTA_ while age was borderline significantly correlated with TAI_Venous_ phase and TAI_Delayed_ (Table 1). These parameters and their corresponding interaction terms were added in all subsequent modeling. In multivariable modeling including TAI_Arterial_, age, and baseline NIHSS, only age and TAI were independently associated with favorable outcome. In the TiCTA multivariable model (including age and baseline NIHSS), only age and NIHSS were independently associated with favorable outcome. In both the delay phase and venous phase CTA multivariable models, only baseline NIHSS was significant. The AIC and BIC were the lowest for the multivariable model with TAI_Arterial_ (Table 4).

**Table 4:** Multivariable regression analysis of the association of TAI measures with favorable functional outcome

10 HU increase of TAI	Regression analysis				AIC	BIC
	Model 1		Model 2			
	aOR (95% CI)	*p*	aOR ((95% CI)	*p*		
Arterial phase	*1.589(1.04–2.428)*	*0.03*	–	–	234.7	251.7
Venous phase	–	–	1.423(0.559–3.621)	0.59	234.1	251.1
Delayed phase	–	–	1.363(0.437–4.248)	0.46	238.0	255.0
TiCTA	1.216(0.87–1.7)	0.25	–	–	235.0	252.0

Model 1: Age, NIHSS, TAI and TAI*NIHSS; Model 2: Age, NIHSS,TAI and TAI*Age; and *italic p* is significant at the 0.05 level

*TAI* thrombus attenuation increase, *OR* odds ratio, *aOR* adjusted odds ratio, *CI* confidence interval, *p* significance value, *NIHSS* National Institutes of Health Stroke Scale, *HU* Hounsfield unit, *TiCTA* time invariant computed tomography angiography, *AIC* Akaike information criterion, *BIC* Bayesian information criterion

## Discussion

This study validates findings from our previous studies that thrombus perviousness is a significant independent predictor of clinical outcome in patients with acute ischemic stroke. Interestingly, our analysis also shows that thrombus perviousness assessment on multiphase CTA either on late phases or by creating a temporal maximum intensity projection does not improve the ability to predict clinical outcome at 90 days or clinical deficit on admission when compared to arterial single-phase CTA.

The relation of TAI with functional outcome has been shown in two other populations in previous studies [4, 5]. Although we found similar associations, the median thrombus attenuation increase we measured was much higher than previously reported. Moreover, the patient outcome in this population is better compared to the Multicenter Randomized Clinical trial of Endovascular treatment of acute ischemic stroke in the Netherlands (MR CLEAN) and Dutch Acute Stroke Study (DUST) populations. The higher TAI could be originated from the contrast agent used, or from the voltage of the X-ray tube. However, these potential sources of TAI measurement bias have been previously studied and have shown not to significantly influence on the perviousness measures in that population of that study [4]. The higher TAI in this study could also be because of the use of 5-mm slice thickness NCCT instead of thin slice NCCTs. Previous studies had assessed TAI on < 2.5 mm reconstruction NCCTs. Partial volume effect in thick slice reconstructions can reduce thrombus attenuation values, thereby resulting in higher TAI values. Nonetheless, our analysis shows that the methodology of measurement of TAI and its ability to predict clinical outcomes are robust across varying NCCT slice thickness.

As previously found [5], in this study, patients with a high TAI have lower baseline NIHSS. This may be because pervious thrombi allow occult anterograde flow to some degree, which may result in less severe ischemia in affected brain [17]. It was also previously shown [4] that despite failure to recanalize, patients with a permeable thrombus were also associated with favorable functional deficits on follow-up.

Venous-phase CTA and TiCTA are conceptually better at visualizing delayed flow through collaterals [6, 11]. It is therefore intuitive to assume that these delayed or time resolved images may also be better at detecting thrombus perviousness. Nonetheless, our analysis shows that TAI measured in the arterial phase is better associated with clinical state. In our opinion, an arterial-phase TAI grasps highly permeable thrombus with fast blood flow in contrary to venous or delayed phases that would grasp delayed arrival of contrast to the clot due to less permeable thrombi or stagnant flow. However, early antegrade flow is a known important predictor of clinical outcome [10, 17, 18]. Therefore, TiCTA only reflects the total thrombus perviousness whilst overlooking if the flow is early or late. Such that, this ability to discriminate fast and medium flowing blood through a permeable thrombus from multiphase CTA could be useful as it may influence the likelihood of recanalization (spontaneous, with intravenous recombinant tissue-type plasminogen activator treatment (IV-rtPA) treatment [5] or with mechanical debunking [19]) and may be linked to the functional clot length and therefore hence an associated chance of filling of perforators and other collateral pathways. We did not test the association of thrombus perviousness with early reperfusion because of heterogeneity of treatment offered and the limited size for the potential patient subgroups; nonetheless, we have shown robust association with relevant clinical outcomes.

Our study has some limitations. Observers placed the region of interest on one single-phase series rather than on all phases separately. This may have resulted in inaccuracies in the region of interest placement of other phases due to suboptimal registration. However, all registrations were checked for accuracy and corrected if necessary. Furthermore, as mentioned above, the thick slice NCCT used in the assessment may have biased the perviousness measurements. Finally, the measurements have been performed by only a single observer per patient. Ideally, all cases should have been reviewed by both users to assess the interobserver variation. However, it was shown that placing three regions of interest is less sensitive to observer variability [14].

Recent guidelines encourage the development of accurate imaging biomarkers to select patients that will likely benefit from intravenous or from intra-arterial treatment [20]. Thrombus permeability is a potentially important biomarker that can be used for patient stratification in a clinical acute setting [4]. This study suggests that TAI measured on arterial-phase CTA alone is a robust predictor of thrombus permeability in patients with acute ischemic stroke.

## Conclusion

Surprisingly, dynamic CTA imaging has little additional benefits in the current assessment of thrombus perviousness. Time invariant CTA was not stronger associated with functional outcome compared to conventional arterial-phase CTA. This suggests that conventional arterial phase CTA is sufficient in the assessment of the thrombi perviousness and that the speed of contrast passage is important for the functional outcome of patients with acute ischemic stroke.

## Abbreviations

AIS: Acute Ischemic Stroke
TiCTA: time-invariant CTA
MCTA: multiphase CTA
HU: Hounsfield Unit
TAI: Thrombus Attenuation Increase
NIHSS: National Institutes of Health Stroke Scale
AUC: receiver operating areas under the curves
TAI_Arterial_: Thrombus Attenuation Increase on the Arterial Phase
TAI_Venous_: Thrombus Attenuation Increase on the Venous Phase
TAI_Delayed_: Thrombus Attenuation Increase on the Delayed Phase
TAI_TiCTA_: Thrombus Attenuation Increase on the TiCTA
IQR: interquartile range
AIC: Akaike information criterion
BIC: Bayesian information criterion
IV-rtPA: intravenous recombinant tissue-type plasminogen activator treatment

## References

[1] Yoo AJ, Pulli B, Gonzalez RG (2012) Imaging-based treatment selection for intravenous and intra-arterial stroke therapies : a comprehensive review. NIH Public Access 9(7):857–876. 10.1586/erc.11.56 10.1586/erc.11.56 PMC3162247 21809968

[2] Riedel CH, Zimmermann P, Jensen-Kondering U, Stingele R, Deuschl G, Jansen O (2011) The importance of size: successful recanalization by intravenous thrombolysis in acute anterior stroke depends on thrombus length. Stroke 42(6):1775–1777. 10.1161/STROKEAHA.110.609693 10.1161/STROKEAHA.110.609693 21474810

[3] Topcuoglu M (2014) A, Arsava EM, Kursun O, Akpinar E, Erbil B. The utility of middle cerebral artery clot density and burden assessment by noncontrast computed tomography in acute ischemic stroke patients treated with thrombolysis. J Stroke Cerebrovasc Dis 23(2):e85–e91. 10.1016/j.jstrokecerebrovasdis.2013.08.026 10.1016/j.jstrokecerebrovasdis.2013.08.026 24119367

[4] Santos EMM, Marquering HA, den Blanken MD et al (2016) Thrombus Permeability Is Associated With Improved Functional Outcome and Recanalization in Patients With Ischemic Stroke. Stroke. 10.1161/STROKEAHA.115.011187 10.1161/STROKEAHA.115.011187 26846859

[5] Santos EMM, Dankbaar JW, Treurniet KM, et al (2016) Permeable thrombi are associated with higher IV-rtPA treatment success in acute ischemic stroke patients. Stroke 47:2058–2065. 10.1161/STROKEAHA.116.013306 10.1161/STROKEAHA.116.013306 27338928

[6] Frölich AMJ, Schrader D, Klotz E (2013). 4D CT angiography more closely defines intracranial thrombus burden than single-phase CT angiography. AJNR Am J Neuroradiol.

[7] Gratama van Andel HAF, Venema HW, Majoie CB, Den Heeten GJ, Grimbergen CA, Streekstra GJ (2009) Intracranial CT angiography obtained from a cerebral CT perfusion examination. Med Phys 36(2009):1074–1085. 10.1118/1.3078043 10.1118/1.3078043 19472612

[8] Konstas AA, Goldmakher GV, Lee TY, Lev MH (2009) Theoretic basis and technical implementations of CT perfusion in acute ischemic stroke, part 1: Theoretic basis. Am J Neuroradiol 30:662–668. 10.3174/ajnr.A1487 10.3174/ajnr.A1487 PMC7051780 19270105

[9] Smit EJ, Vonken E, van der Schaaf IC (2012). Timing-Invariant Reconstruction for Deriving High-Quality CT Angiographic Data from cerebral CT perfusion data. Radiology.

[10] Frölich AMJ, Psychogios MN, Klotz E, Schramm R, Knauth M, Schramm P (2012) Antegrade flow across incomplete vessel occlusions can be distinguished from retrograde collateral flow using 4-dimensional computed tomographic angiography. Stroke 43:2974–2979. 10.1161/STROKEAHA.112.668889 10.1161/STROKEAHA.112.668889 22961960

[11] Menon BK (2015) D ‘esterre CD, Qazi EM, et al. multiphase cT angiography: a new tool for the imaging triage of patients with acute ischemic stroke 1. Radiology 275(2):510–520. 10.1148/radiol.15142256 10.1148/radiol.15142256 25633505

[12] Fahmi F, Marquering HA, Borst J et al (2014) 3D movement correction of CT brain perfusion image data of patients with acute ischemic stroke. Neuroradiology. 10.1007/s00234-014-1358-7 10.1007/s00234-014-1358-7 24715201

[13] Klein S, Staring M, Murphy K, Viergever MA, Pluim JPW (2010). Elastix: a toolbox for intensity-based medical image registration. IEEE Trans Med Imaging.

[14] Santos EMM, Yoo AJ, Beenen LF et al (2015) Observer variability of absolute and relative thrombus density measurements in patients with acute ischemic stroke. Neuroradiology. 10.1007/s00234-015-1607-4 10.1007/s00234-015-1607-4 PMC4773501 26494462

[15] Informatik (2009) Im Focus das Leben, Beiträge der 39. Jahrestagung der Gesellschaft für Informatik e.V. (GI), 28.9.-2.10.2009, Lübeck, Proceedings

[16] Hoaglin DC, Iglewicz B, Tukey JW (1986). Performance of some resistant performance rules for labeling outlier. J Am Stat Assoc.

[17] Ahn SH, d’Esterre CD, Qazi EM et al (2015) Occult anterograde flow is an under-recognized but crucial predictor of early recanalization with intravenous tissue-type plasminogen activator. Stroke 46(4):968–975. 10.1161/STROKEAHA.114.008648 10.1161/STROKEAHA.114.008648 25700286

[18] Labiche LA, Malkoff M, Alexandrov AV (2003) Residual flow signals predict complete recanalization in stroke patients treated with TPA. J Neuroimaging 13(1):28–33. 10.1177/1051228402239714 12593128

[19] Chueh JY, Wakhloo AK, Hendricks GH, Silva CF, Weaver JP, Gounis MJ (2011) Mechanical characterization of thromboemboli in acute ischemic stroke and laboratory embolus analogs. Am J Neuroradiol 32(7):1237–1244. 10.3174/ajnr.A2485 10.3174/ajnr.A2485 PMC7966072 21596804

[20] Jauch EC, Saver JL, Adams HP et al (2013) Guidelines for the early management of patients with acute ischemic stroke: a guideline for healthcare professionals from the American Heart Association/American Stroke Association. Stroke 44(3):870–947. 10.1161/STR.0b013e318284056a 10.1161/STR.0b013e318284056a 23370205

